# Detecting lies in investigative interviews through the analysis of response latencies and error rates to unexpected questions

**DOI:** 10.1038/s41598-024-63156-y

**Published:** 2024-05-28

**Authors:** Giulia Melis, Martina Ursino, Cristina Scarpazza, Andrea Zangrossi, Giuseppe Sartori

**Affiliations:** 1 Department of General Psychology, University of Padua, Padova, Italy; 2 Human Inspired Technology Research Centre, University of Padua, Padova, Italy; 3 Translational Neuroimaging and Cognitive Lab, IRCCS San Camillo Hospital, Venice, Italy; 4 Padova Neuroscience Center (PNC), University of Padua, Padova, Italy

**Keywords:** Psychology, Human behaviour

## Abstract

In this study, we propose an approach to detect deception during investigative interviews by integrating response latency and error analysis with the unexpected question technique. Sixty participants were assigned to an honest (n = 30) or deceptive group (n = 30). The deceptive group was instructed to memorize the false biographical details of a fictitious identity. Throughout the interviews, participants were presented with a randomized sequence of control, expected, and unexpected open-ended questions about identity. Responses were audio recorded for detailed examination. Our findings indicate that deceptive participants showed markedly longer latencies and higher error rates when answering expected (requiring deception) and unexpected questions (for which premeditated deception was not possible). Longer response latencies were also observed in participants attempting deception when answering control questions (which necessitated truthful answers). Moreover, a within-subject analysis highlighted that responding to unexpected questions significantly impaired individuals’ performance compared to answering control and expected questions. Leveraging machine-learning algorithms, our approach attained a classification accuracy of 98% in distinguishing deceptive and honest participants. Additionally, a classification analysis on single response levels was conducted. Our findings underscore the effectiveness of merging response latency metrics and error rates with unexpected questioning as a robust method for identity deception detection in investigative interviews. We also discuss significant implications for enhancing interview strategies.

## Introduction

Forensic psychologists have developed various methodologies to distinguish between truth and deception under controlled experimental conditions. Notable examples include the Concealed Information Test (CIT)^1^ and the autobiographical Implicit Association Test (aIAT)^2^. Despite these advancements, there is a noticeable gap in the development of methods for assessing statements’ credibility during investigative interviews. Ideally, these methods should be integrated smoothly into the flow of the investigative interview and remain imperceptible to the subject under examination, thereby avoiding any explicit indication of credibility assessment^3^. Current techniques, such as the CIT, the aIAT, and traditional polygraph testing, tend to make the subjects overtly aware of the evaluation process, potentially affecting their responses. An attempt to address these limitations has been recently made by combining these behavioral techniques with eye tracking^4,5^, given the relation between eye movements and endogenous brain dynamics^6,7^. However, using the aIAT or similar tools is not always applicable in police procedures or legal systems. This paper introduces a methodology that relies on the analyses of audio recordings of investigative interviews to accurately distinguish between deceptive and honest responses. Specifically, participants were divided into two groups (honest or deceptive) and subjected to control, expected, or unexpected open-ended questions regarding their identity (true or fabricated). The responses were audio recorded, and the response latencies and the number of errors the two groups made in response to the three types of questions were analyzed and compared.

### Cognitive load and interview techniques

A multitude of studies have suggested that deception is cognitively more demanding than truth telling, given the additional cognitive processes engaged during lying^8–14^. One metric extensively used to probe these cognitive demands is response latencies. For instance, whereas truthful responses generally occur within 400 ms^15,16^, deceptive responses are typically associated with longer response latencies^8,15,17,18^. This difference in response latencies is thought to reflect cognitive load^19^, hinting that longer response durations could be pivotal in distinguishing deception from honesty.

It is worth noting, however, that the cognitive demands of lying are not uniform. Whether challenging to fabricate or relatively straightforward, the lie’s complexity plays a role in this variability^20^. Additionally, individuals vary in their proficiency at lying^21^, with many deceivers rehearsing their deception in advance^22^. In the deception detection landscape, a clear strategy divergence is naturally observed between truth tellers and deceivers^23^. Truth tellers rely on authentic memories, and the most commonly employed strategy is to *“narrate the events exactly as they occurred,”* reflecting the underlying belief that *“when one is innocent, there is little need to manipulate the narrative to enhance its credibility.”* This prevalent conviction is anchored in the illusion of transparency^24^, according to which *“the innocence shows on the outside and the truth will come out.”* Truth tellers choose not to employ explicit strategies; therefore, they only must recall what they truly experienced when answering investigative questions, relying on their memory. This approach, however, also has a significant consequence in that truth tellers do not always report everything they can remember. To overcome this limitation, Porter et al.^25^ designed the asymmetric-information management instruction, which informs interviewees, among other things, that more detailed statements can be more easily classified as genuine or fabricated. The purpose of this strategy is to encourage truth tellers to be more verbally forthcoming and to persuade deceivers to be more verbally reserved. In their study, this technique improved the classificatory performance of discriminant analysis because truth tellers provided (and deceivers concealed) more information.

In contrast to truth tellers, who can solely rely on recalling their genuine experiences, deceivers tend to script their narratives in advance^22^. This training allows them to rely on memory during questioning, thus reducing cognitive overhead. Consequently, they often stick to core story elements and avoid incorporating unconventional details^26^. Given a choice, deceivers tend to use actual events over fabrications to reduce cognitive effort^27^. This strategy helps them maintain narrative coherence and avoid arousing suspicion. Intriguingly, premeditation significantly alters the cues indicative of deceit and associated response latencies. Well-crafted lies often show reduced response latencies compared to spontaneous deceptions^16,26,28,29^. This intricacy in deception presents significant challenges for detectors, especially when they are confronted with carefully crafted lies^30^.

Due to the potential of cognitive load, there has been a significant shift in research focus to enhance deception detection by highlighting signs of cognitive strain^31–36^. This is achieved by amplifying the cognitive load on respondents using several innovative techniques. For instance, respondents might be asked to narrate events in reverse order^35,37^, maintain uninterrupted eye contact during interviews^38^, or complete an additional task. This exploits the dual-task paradigm, in which juggling two simultaneous tasks can be particularly taxing^39–42^.

Task switching is a crucial high-level cognitive ability that enables individuals to direct and allocate their attentional resources across multiple sequential cognitive tasks^43^. In typical deception paradigms, task switching occurs even when one transitions from truth telling to lying or vice versa, such as when the control and target questions are presented randomly. The individual engaged in deceit must switch between these two cognitive tasks, thereby influencing task performance^44^. In the task-switching literature, the well-established presence of a performance switch cost is evidenced by participants being slower and more error-prone when switching tasks than when repeating the same task^45–47^. Therefore, exploring switch costs in task switching is a potential, albeit underexplored, avenue for lie detection^44^.

A second technique that has been developed relates to the unexpectedness of the questions asked. The strategy of reducing cognitive load by preparing for an interview—anticipating potential questions and formulating answers in advance—is effective only if the deceiver accurately predicts the questions that will be asked. In other words, this strategy falters when the interviewer poses questions the deceiver has not anticipated^48^. From this perspective, the “unexpected-question technique” can be fruitfully implemented in the investigative interview to enhance lie detection accuracy. When faced with unanticipated questions, truth tellers respond instinctively, whereas deceivers struggle to fabricate answers without rehearsed responses^49^. Such questions can span a range of topics, from planning future behavioral intentions, such as *“I want you to think back to when you planned your errand. I want you to tell me about your planning, and I want you to be as detailed as possible”*^50,51^ and spatial–temporal details and shifts, e.g., *“In relation to the front door, where did you and your friend sit?”*^40,49,52^ to specific information from identification cards, such as *“Is Venezia the chief town of your residence region?”*^53^. The main benefit of using these questions is that they effectively disrupt a deceiver’s narrative. To differentiate between truth tellers and deceivers, their response latencies to unexpected questions are compared to responses to verifiable control questions, such as *“Are you in front of a computer?”*^48,54,55^. These answers to control questions serve as a baseline for genuine response latencies, highlighting that deceivers typically take longer to respond to unexpected questions^53^. This technique’s efficacy is well supported^40,50–52,56,57^, demonstrating a 71% success rate in distinguishing truth from deceit. This marks a significant improvement over the 56% success rate achieved by conventional methods^58^.

In light of this overview and in the framework of our research, we have adopted these strategies to explore how individuals engage in deception versus truth telling. Specifically, we employed an experimental design that leverages task switching and unexpected questions. In this experiment, participants were asked to alternate their responses among unexpected, expected, and control questions. Notably, participants in the deceptive group were required to respond truthfully to control questions, whereas deceptive responses were solicited for the other two types of questions. The aim was to assess the cognitive load imposed by the requirement to lie, exploiting the discrepancy between providing truthful responses and fabricating false ones. Including unexpected questions amplifies the mental effort required for deception, thus accentuating the disparity in accuracy and response latencies between lying and truth telling.

### Identity deception

The main focus of the present study is to evaluate a lie detection method that integrates response latency analysis and the unexpected-question technique to identify a specific form of lying: identity deception. Identity deception^59^ is a distinct form of deception wherein individuals intentionally conceal their identity, impersonate another individual, or use counterfeit identity documents, undermining the credibility of identity information through deliberate deceit. Alarmingly, this type of deceit is widespread. For example, in a case study^60^, it was found that nearly a third of police suspects adopted false identities, predominantly by modifying their names. The advent of the digital era has magnified this challenge, with frequent alterations observed in attributes such as images, names, age, and gender ^61,62^. Drouin et al.^63^ found that only between 16 and 32% of participants maintained consistent honesty across websites, with a scant 0.2% believing that others were universally truthful online. Regrettably, online identity deception can also arise from malevolent motives. A particularly disconcerting manifestation of this deceit can be seen in heinous activities, such as child grooming^64^.

On a large scale, identity deception poses a critical threat to national security. Forged documents provide terrorists and criminals with an avenue to bypass security protocols, a reality underscored by the proliferation of counterfeit documents at Europe’s borders^65^ and incidents such as the Twin Towers bombing and the more recent attack on the Brussels Maelbeek metro on March 22, 2016, in which a terrorist used several false identities to pass through multiple states. At the time of his transit through Italy, for example, he entered the country under the name of a former Inter Milan football player^66^. Various criminal activities, including terrorism, human and drug trafficking, and money laundering, often involve counterfeit identities^67,68^. The 2021 EU Organized Crime Report highlights that technological advancements have facilitated the ease with which counterfeit documents can be procured^69^. In light of this information, it is evident that deceptive identities are becoming an increasingly pervasive issue. The rapid evolution of these false identities accentuates the pressing need for practical solutions to detect and counter identity deception.

Researchers have recently employed techniques such as the unexpected-question method to detect identity deception. Jupe et al.^70^ investigated whether contextual and perceptual details and language use could effectively differentiate identity deceivers from truth tellers in response to outcome questions (related to the outcome of an event) and unexpected process questions (specific to the planning phase or the progression experience of an event). Contrary to expectations, the findings suggested that contextual and perceptual details may not have diagnostic value when applied to the cross‐situational domain of identity deception. However, significant differences were found in the way that truth tellers and deceivers manage their overall verbal outputs regarding positive emotion language and cognitive process words. In particular, deceivers used more positive language in their interviews overall than truth tellers, whereas truth tellers used more cognitive process words than deceivers. Moreover, process questions were found to cause a greater use of mental processes and causal words than outcome questions, suggesting that they impose a greater cognitive burden on individuals.

In other studies, however, researchers have also used tools such as mouse and keyboard movement analyses to detect identity deception with the unexpected-questions technique^71,72^. In Monaro’s study, the authors required participants to learn and adopt a fictitious identity profile. This was followed by a series of control and target queries, expected and unexpected, about their assumed identities, to which the participants responded via a computerized task. In this task, the authors scrutinized mouse-tracking trajectories. Among various metrics, response latencies and error rates were particularly effective in differentiating between honest and deceptive responses, with deceivers exhibiting slower responses and committing more errors when responding to unexpected questions than truth tellers. Moreover, individuals who were lying exhibited longer response latencies and higher error rates when responding to unexpected questions compared to expected ones^53^.

These methods differ significantly from those that require prior knowledge of an individual’s identity, such as the aIAT^2^ and the RT-CIT. In many real-world scenarios, examiners might not have access to comprehensive identity information, rendering specific methods less practical. Furthermore, patterns in mouse and keyboard movements can be seen as implicit behavioral indicators; individuals engaging in a computer-based task are often unaware that these specific patterns are being observed and analyzed^73^. Studies suggest that unintentional behaviors are among the most reliable indicators of deception^74^, underscoring the potential of covert deception detection. In an investigative interview context, the subject remains unaware of the objective of detecting deception and the specific metrics under observation. The subject’s lack of control over metrics, such as response latencies in responding to unexpected questions, increases the technique’s effectiveness.

### The present study

To the best of our knowledge, this is the first study conducted to explore the combined efficacy of the unexpected-question technique, response latency analysis, and error measurements in detecting identity deception during investigative interviews. A distinctive contribution of this study is its application in the context of investigative interviews, a setting in which investigators routinely and extensively use audio recordings. Notably, the analysis of these recordings has traditionally been focused primarily on the verbal content of the interviewee’s responses, thereby neglecting a detailed examination of response latencies^37,38,52,56^. Although interviewees might be aware of the recording procedure, they cannot anticipate the in-depth analysis of the timing of their responses to specific queries. These response latencies serve as implicit indicators, providing data less prone to intentional manipulation by the interviewee.

This approach has three main advantages. First, it allows for lie detection without the individual’s awareness that the technique is focused on the credibility assessment, thereby ensuring that the continuity of investigative interviews is not compromised; second, it can be potentially applied retrospectively to interviews that have been conducted, allowing for exploitation of data that are routinely acquired; third, it does not impact standard procedures of the judicial or police system because no ad hoc tests are administered. Moreover, compared to transcripts and audio-visual lie detection, detecting lies via audio recordings may provide a clearer and more reliable source of information. Research has shown that the ability to discriminate between lies and truths is notably weaker when one relies on video presentations rather than written transcripts. Furthermore, messages tend to be perceived as less truthful when judged through video than through audiovisual or purely audio presentations. Finally, deception detection accuracy diminishes when assessments are made using video, with individuals demonstrating greater precision in identifying falsehoods through auditory cues rather than visual ones^75^.

The primary objective of this study was to investigate unexpected questions’ effects on information reconstruction, comparing response latencies and error rates between the two groups: those responding honestly and those engaging in deception. Specifically, the study manipulated two main factors: the nature of the questions (control vs. expected vs. unexpected) and the participant’s veracity (honest vs. deceptive). As dependent variables, we measured the participants’ response latencies in responding to the questions and the error rates in their answers to evaluate these manipulations’ effects.

We hypothesized that:

1. deceptive participants would exhibit longer response latencies when responding to expected and unexpected questions than honest participants due to the increased cognitive load required to lie;
2. deceptive participants would exhibit higher error rates when responding to expected and unexpected questions than honest participants due to the increased cognitive load required to lie;
3. in a) the deceptive and b) the honest groups, answering unexpected questions would produce longer response latencies than answering control and expected questions due to the additional challenge of adjusting premeditated answers on the spot;
4. in a) the deceptive and b) the honest groups, answering unexpected questions would produce higher error rates than answering control and expected questions due to the additional challenge of adjusting premeditated answers on the spot;
5. answering the unexpected questions would be perceived as a) more challenging and b) more unanticipated with respect to the other question types.

## Materials and methods

### Participants

The study sample comprised 60 native Italian speakers recruited through the experimenter’s network and word of mouth. An a priori power analysis revealed that a sample size of 60 is sufficiently large to achieve a statistical power of (1-β) = 0.95 in a mixed ANOVA (within-between interaction) involving two groups, given a significance level (α) of 0.05 and an effect size of 0.25. This sample size is consistent with other lie detection research based on response latencies that employed a similar experimental design^53,71,76^.

All participants were university students, 34 females (57%) and 26 males (43%) aged between 21 and 29 (M = 23.68, SD = 1.32), with educational backgrounds ranging from 16 to 19 years (M = 17.82; SD = 0.59). The two experimental groups were statistically homogeneous in terms of age, education, and gender distribution, ensuring comparability in subsequent analyses.

No external incentives (monetary rewards or university credits) were provided. Participants were randomly assigned to one of two experimental conditions: half (30 participants) were placed in the honest group and instructed to answer interview questions (control, expected, and unexpected) truthfully using their personal information. The other half were in the deceptive group and directed to respond with false details consistent with a fictitious identity provided to them.

### Experimental procedure

Ethical approval was obtained from the Ethics Committee at the Department of General Psychology, University of Padua. All methods were performed in accordance with the Declaration of Helsinki. Participants were informed about the confidentiality of their data and their right to withdraw at any time without repercussions. Informed consent was obtained from all participants. All materials provided to participants were in Italian, and the interviews were conducted in Italian.

Participants in the honest group initially completed a file with their details: name, surname, date of birth, place of birth, email, the first six letters of their tax code, place of residence, address, phone number, completed three-year degree course, and the year they obtained their three-year degree. Conversely, participants in the deceptive group were given five minutes to memorize a fictitious identity with the same information categories. Specifically, two identities to be memorized were provided to the deceptive group: one male (assigned to male deceivers) and one female (assigned to female deceivers). The complete instructions given to the participants and details on the identities are fully reported in the Supplementary Material (S1). Following this, the honest and deceptive groups performed five arithmetic tasks. This procedure, consisting of rehearsals with breaks and distraction tasks in between, facilitates the retention of the false identity^53^.

Participants then orally recounted their details. Those in the deceptive group only advanced to the next phase upon accurately recalling their assigned identity. If inaccuracies occurred, they reviewed the details for another five minutes, completed another set of arithmetic tasks, and attempted recall again. During this phase, only a few deceptive participants recounted their details twice. Consequently, the number of repetitions ranged from a minimum of one, achieved by all participants, to a maximum of two.

Each participant underwent a standardized, face-to-face interview of 36 open-ended questions, detailed in the Supplementary Material (S2). Interviews lasted approximately five minutes and began with a reminder that questions pertained to previously provided personal information. The questions were categorized as 12 control, 12 expected, and 12 unexpected items and were presented in a randomized order. All participants received the questions in the same order, as detailed in the Supplementary Material (S2). Control questions concerned the experiment’s context or verifiable physical attributes, allowing interviewers to verify responses directly (e.g., *“In which month are we currently?”*). Response latencies to these questions established a benchmark for the typical duration required for a participant to answer truthfully. Expected questions were based on information directly supplied in the file (e.g., *“In which month were you born?”*). Honest and deceptive participants could readily access answers from their genuine personal details or the memorized fictitious information. On the contrary, unexpected questions probed for information not explicitly documented or recalled but inferred from primary details (e.g., *“What is your zodiac sign?”*). Honest participants would respond based on their knowledge, whereas deceptive participants had to fabricate responses on the spot. For example, an individual born on September 22nd would intuitively recognize their zodiac sign as Virgo. Similarly, a resident of Padua would know its regional capital. In contrast, deceivers, not well acquainted with their assigned false identities, faced the challenge of quickly deducing the correct answer from their fabricated data.

All sessions were audio recorded using the iPhone 13’s Voice Memo app (iOS 17) to collect response latencies, the precise time gap between the conclusion of the interviewer’s question and the onset of the participant’s response, and errors. Post-interview, participants completed two Google Forms questionnaires to assess the perceived difficulty in answering each question (rated on a Likert scale from 1 *“very easy”* to 5 *“very difficult”*) and the level of anticipation (rated on a Likert scale from 1 *“slightly anticipated”* to 3 *“highly anticipated”*) for expected and unexpected questions. The total duration of the experiment was approximately 40 min. At the end of the session, participants provided written consent to use their data.

### Data analysis

The Audacity software (www.audacityteam.org) was employed to extract response latencies, providing precision at the millisecond level. The latencies were measured from the end of the interviewer’s question to the onset of the interviewee’s first meaningful word, excluding fillers such as *“umm”*.

Errors were categorized as responses that did not align with the participant’s personal information (whether true or false) and responses in which the participant claimed not to know the correct answer. As for the honest group, their responses were cross-referenced with the information that the participants were asked to provide by filling out the form before the interview. From this information, it was possible to code errors by comparing the responses of the honest participants (audio-recorded) with the information provided on the completed form. As for the deceivers, if they provided information different from what they were instructed to memorize, the response was considered an error. If a participant fabricated information other than what they were supposed to recall, this was also considered an error and analyzed accordingly. Control questions were coded instantaneously by the single interviewer during the sessions or, where necessary, immediately after the interview through the review of audio recordings. The responses to expected and unexpected questions were coded post-interview by the interviewer and an additional researcher through the review of audio recordings. A coding system was employed where a score of "0" signified alignment between the participant's response and the predefined information (correct response), while a score of "1" indicated a discrepancy (incorrect response). In assessing inter-rater reliability, the analysis revealed direct agreement between the interviewer and the additional researcher in 97% of the cases. Additionally, Cohen's kappa^77^ was used to measure the accuracy and consistency of the ratings assigned by the two researchers to score errors in answers to expected and unexpected questions. The kappa statistic was 0.868, indicating almost perfect agreement between the raters^78^.

Data consisted of repeated responses from individual participants, with each participant answering control, expected, and unexpected questions. A mixed ANOVA with a 2 (veracity: honest vs. deception) × 3 (question type: control vs. expected vs. unexpected) design was conducted to analyze the response latencies and error rates. This analysis assessed the main effects and interaction between veracity, a between-subjects factor, and question type, a within-subjects factor, on the measured outcomes.

To further examine the veracity’s impact (honest vs. deceptive), a between-subjects analysis was implemented by conducting an independent-samples *t*-test. Additionally, to explore the specific impact of question type (control, expected, and unexpected) in each group, two separate repeated-measures ANOVAs—one for the honest group and one for the deceptive group—were conducted.

The same analysis was conducted to ascertain the influence of veracity and type of question on the perceived difficulty encountered and the participants’ anticipation in answering the questions. These results were extracted from the two final questionnaires. All analyses were conducted using Jamovi^79^.

We further examined the feasibility of classifying individual subjects under the two groups (honest or deceptive), quantifying this classification’s accuracy. For this aim, we trained and validated a series of machine-learning classification models through a tenfold cross-validation procedure using PyCaret, an open-source low-code machine-learning library implemented in Python (https://pycaret.org). With PyCaret, various classifiers with different accuracies are produced, as shown in the result section.

The use of machine learning in the forensic classification of individual subjects is promising and significant. Traditional analyses typically rely on statistical methods that compare two or more groups to discern patterns or differences. Although statistical analyses provide a comprehensive view of group differences, they fall short in accurately classifying individual subjects. In contrast, machine learning offers an approach that can uncover intricate patterns and relationships conventional statistical methods often overlook. This precision is especially vital in real-world forensic settings, where individual accuracy at the single-subject level is required^80^.

## Results

### Latency analysis

A mixed ANOVA was conducted to examine the impact of veracity (honest vs. deceptive) and question type (control, expected, unexpected) on response latencies. The analysis demonstrated significant main effects for veracity, F(1, 58) = 77.7, *p* < 0.001, η^2^ = 0.172; type of question, F(2, 116) = 190.9, *p* < 0.001, η^2^ = 0.400; and their interaction, F(2, 116) = 84.9, *p* < 0.001, η^2^ = 0.178, suggesting that both factors and their interaction significantly predict response latencies. To explore the simple main effect of veracity in each question type, an independent-samples *t*-test (between-subjects analysis) was conducted (Table 1). These results confirm our Hypothesis 1.

**Table 1:** Descriptives for response latencies and independent t-test results.

Question type	Honest		Deceptive		*t*(58)	*p*	Cohen's *d*
	*M*	*SD*	*M*	*SD*			
Control	0.82	0.39	1.21	0.48	− 3.38	.001	− 0.87
Expected	0.77	0.44	1.36	0.61	− 4.27	< .001	− 1.10
Unexpected	1.72	0.76	5.90	2.26	− 9.60	< .001	− 2.48

Additionally, two repeated-measures ANOVAs (one for each group) were conducted to examine the simple effect of question type on response latencies, with question type as the within-subjects factor. The analysis of the deceptive group revealed a significant effect of question type, F(2, 58) = 147, *p* < 0.001, η^2^ = 0.721, indicating a substantial variance in response latencies based on the type of question presented. A post hoc analysis with Bonferroni correction highlighted significant differences in the latencies between responses to control vs. unexpected questions (difference = − 4.696, SE = 0.3908, *t* = − 12.02, *p*_*bonferroni*_ < 0.001) and between response latencies to the expected and unexpected questions (difference = − 4.544, SE = 0.3655, *t* = 12.43, *p*_*bonferroni*_ < 0.001). In both instances, the response latencies for the unexpected questions were notably higher. We found no differences between the control and expected-question conditions (difference = − 0.152, SE = 0.0665, *t* = − 2.29, *p*_*bonferroni*_ = 0.089). These results confirm our Hypothesis 3a.

**Figure 1:**
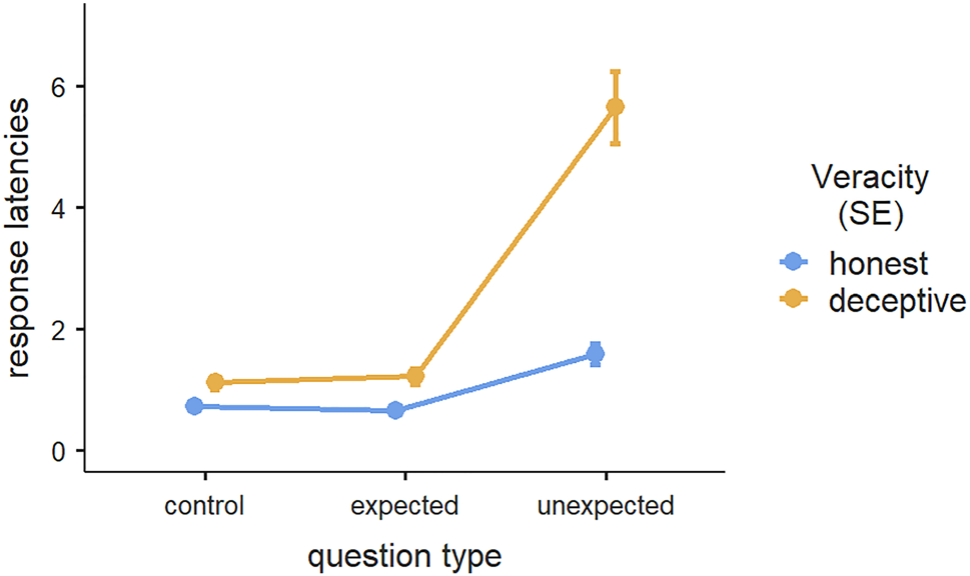
Interaction effects of response latencies (in seconds) for honest and deceptive participants across control, expected, and unexpected questions. Error bars displayed in this chart represent the standard error.

We also observed the same pattern in the honest group, thus confirming our Hypothesis 3b. The analysis revealed a significant effect of question type, F(2, 58) = 54.3, *p* < 0.001, η^2^ = 0.388. A post hoc analysis with Bonferroni correction highlighted significant differences between response latencies to the control and unexpected questions (difference = − 0.8986, SE = 0.1233, *t* = − 7.29, *p*_*bonferroni*_ < 0.001) and between response latencies to the expected and unexpected questions (difference = − 0.9511, SE = 0.1228, *t* = 7.74, *p*_*bonferroni*_ < 0.001). In both instances, the latencies when answering to the unexpected questions were notably higher. We found no differences between the response latencies to control and expected-question types (difference = 0.0526, SE = 0.0355, *t* = 1.48, *p*_*bonferroni*_ = 0.450). Please refer to Fig. 1 for a graphic representation of the interaction effects between groups’ response latencies.

### Error analysis

A mixed ANOVA was conducted to examine the impact of veracity (honest vs. deceptive) and question type (control, expected, unexpected) on error rate. The analysis indicated a significant main effect for veracity, F(1, 58) = 91.1, *p* < 0.001, η^2^ = 0.144; question type, F(2, 116) = 151.3, *p* < 0.001, η^2^ = 0.413; and their interaction, F(2, 116) = 71.2, *p* < 0.001, η^2^ = 0.194, suggesting that both factors and their combination significantly predict error rate. The between-subjects analysis was conducted to explore the simple effect for veracity on each question type by conducting an independent-samples *t*-test (Table 2). These results confirm our Hypothesis 2.

**Table 2:** Descriptives for errors and independent t-test results.

Question type	Honest	Deceptive			*t*(58)	*p*	Cohen's *d*
	*M*	*SD*	*M*	*SD*			
Control	0.03	0.18	0.03	0.18	0.00	1.000	0.00
Expected	0.07	0.25	0.60	0.72	− 3.81	< .001	− 0.98
Unexpected	0.87	0.63	4.67	2.14	− 9.34	< .001	− 2.41

Additionally, two repeated-measures ANOVAs (one for each group) were conducted to examine the simple effect for question type on error rates, with question type as the within-subjects factor. The analysis of the deceptive group revealed a significant effect of question type, F(2, 58) = 118, *p* < 0.001, η^2^ = 0.720, indicating a substantial error variance based on the type of question presented. A post hoc analysis with Bonferroni correction highlighted significant differences between error rates when answering to the control and unexpected questions (difference = − 4.633, SE = 0.391, *t* = − 11.85, *p*_*bonferroni*_ < 0.001) and between error rates in response to the expected and unexpected questions (difference = − 4.067, SE = 0.389, *t* = 10.45, *p*_*bonferroni*_ < 0.001). In both instances, the number of errors when answering to the unexpected questions was notably higher. These results confirm our Hypothesis 4a. Moreover, a significant difference also emerged when we compared the answers to control and expected questions (difference = − 0.567, SE = 0.141, *t* = − 4.01, *p*_*bonferroni*_ = 0.001).

**Figure 2:**
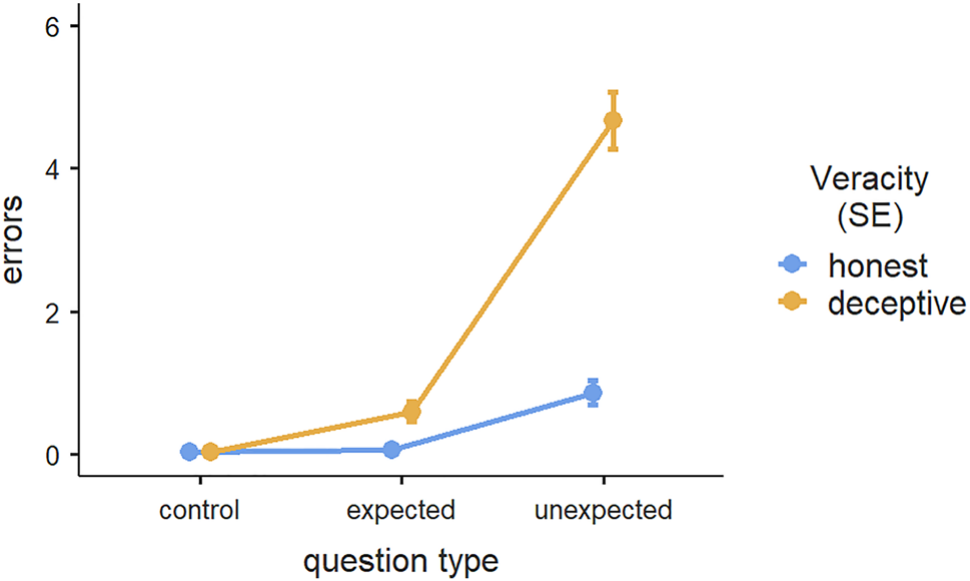
Interaction effects of error rates for honest and deceptive participants across control, expected, and unexpected questions. Error bars displayed in this chart represent the standard error.

The analysis of the honest group revealed a significant effect of question type, F(2, 58) = 41.6, *p* < 0.001, η^2^ = 0.483. A post hoc analysis with Bonferroni correction highlighted significant differences in errors between the responses to control and unexpected questions (difference = − 0.8333, SE = 0.1183, *t* = − 7.047, *p*_*bonferroni*_ < 0.001) and between the answers to expected and unexpected questions (difference = − 0.8000, SE = 0.1213, *t* = − 6.595, *p*_*bonferroni*_ < 0.001). In both instances, the error rates when answering the unexpected questions were higher, thus confirming our Hypothesis 4b. We found no differences between the response errors to control and expected questions (difference = − 0.0333, SE = 0.0584, *t* = -0571, *p*_*bonferroni*_ = 1.000). Please refer to Fig. 2 for a graphic representation of these results.

### Questionnaire analysis

As previously mentioned, participants were instructed to assess each question’s degree of anticipation and perceived difficulty by responding to two questionnaires, the first using a 5-point Likert scale and the second using a 3-point Likert scale.

### Difficulty questionnaire analysis

A mixed ANOVA was conducted to assess the effects of veracity (honest vs. deceptive) and question type (control, expected, unexpected) on perceived response difficulty during the interview. The analysis indicated a significant main effect for veracity, F(1, 58) = 25.4, *p* < 0.001, η^2^ = 0.077, and question type, F(2, 116) = 247.9, *p* < 0.001, η^2^ = 0.546, and a significant interaction effect between the factors, F(2, 116) = 32.6, *p* < 0.001, η^2^ = 0.072. These findings assert that the veracity, the question type, and their interaction significantly help predict perceived difficulty. The between-subjects analysis was conducted to highlight veracity’s simple effect by conducting an independent-samples *t*-test (Table 3).

**Table 3:** Descriptives for difficulty and independent t-test results.

Question type	Honest	Deceptive			t(58)	*p*	Cohen's *d*
	*M*	*SD*	*M*	*SD*			
Control	1.26	0.25	1.21	0.25	0.73	0.471	0.19
Expected	1.26	0.18	1.61	0.49	− 3.67	< .001	− 0.95
Unexpected	1.99	0.37	2.87	0.65	− 6.50	< .001	− 1.68

We also observed significant disparities in the honest and deceptive groups when we conducted an additional repeated-measures ANOVA to examine question type’s simple effect on perceived difficulty in each group. The analysis of the deceptive group revealed a significant effect of question type, F(2, 58) = 136, *p* < 0.001, η^2^ = 0.683, indicating a substantial variance in perceived difficulty based on the type of question presented. A post hoc analysis with Bonferroni correction highlighted significant comparisons between perceived difficulty when answering control and expected questions (difference = -0.397, SE = 0.0975, *t* = − 4.07, *p*_*bonferroni*_ < 0.001), control and unexpected questions (difference = -1.661, SE = 0.1181, *t* = − 14.06, *p*_*bonferroni*_ < 0.001), and between expected and unexpected questions (difference = − 1.264, SE = 0.0988, *t* = − 12.80, *p*_*bonferroni*_ < 0.001).

The analysis of the honest group revealed a significant effect of question type, F(2, 58) = 162, *p* < 0.001, η^2^ = 0.620. A post hoc analysis with Bonferroni correction highlighted a significant comparison between perceived difficulty when answering control and unexpected (difference = − 0.733, SE = 0.0460, *t* = − 16.0, *p*_*bonferroni*_ < 0.001) and between expected versus unexpected questions (difference = − 0.733, SE = 0.0571, *t* = − 12.8, *p*_*bonferroni*_ < 0.001). However, honest participants did not perceive more difficulty in answering expected questions than in answering control questions (difference = 6.67e-11, SE = 0.0355, *t* = 1.88e-9, *p*_*bonferroni*_ = 1.000). The difference observed within the honest and deceptive groups in perceived difficulty when answering unexpected questions compared to answers provided to control and expected questions confirms our Hypothesis 5a.

### Anticipation questionnaire analysis

A mixed ANOVA was conducted to assess the effects of veracity (honest vs. deceptive) and question type (expected vs. unexpected) on perceived response anticipation during the interview. The analysis indicated a significant main effect for veracity, F(1, 58) = 6.16, *p* = 0.016, η^2^ = 0.007, and for question type, F(1, 58) = 637.52, *p* < 0.001, η^2^ = 0.842. We found no significant interaction effect between veracity and question type, F(1, 58) = 3.48, *p* = 0.067, η^2^ = 0.005. These findings assert that veracity and question type help predict perceived anticipation. The descriptive statistics presented in Table 4 indicate that the average anticipation scores for the honest group are consistently lower than those for the deceptive group for both expected and unexpected questions. Furthermore, within both groups, expected questions elicit higher levels of anticipation compared to unexpected ones, thus, confirming our Hypothesis 5b.

**Table 4:** Descriptives for anticipation and independent t-test results.

Question type	Honest		Deceptive	
	*M*	*SD*	*M*	*SD*
Expected	2.64	0.40	2.86	0.12
Unexpected	1.48	0.25	1.50	0.23

### Deception detection at the single-subject level

The concluding analysis was conducted using PyCaret to assess the accuracy of various machine-learning classifiers in distinguishing between honest and deceptive participants. For this classification task, latencies and the number of errors were employed as features. Each was further differentiated into control, expected, and unexpected, resulting in six distinct features. The six features were entered into three machine-learning algorithms: logistic regression, KNN, and random forest classifier. We chose multiple algorithms to prevent the selection of only the highest-performing model by chance, checking whether there is substantial variation in classification accuracy across various classifiers^80^. Moreover, we chose these specific algorithms to test the robustness of our classification performance because they are based on various underlying assumptions. Indeed, when machine-learning models based on fundamentally different principles yield comparable outcomes, it can be reasonably assumed that the results do not rely on particular assumptions.

Leveraging machine-learning models allows for prediction of individual behaviors rather than generalization of a group’s collective behavior^81^. To ensure the out-of-sample generalization, the standard K-fold cross-validation technique (*K* = 10) was employed^80^. This technique is critical, mainly when one works with limited data samples that require out-of-sample accuracy. Through this technique, the data sample was divided into 10 unique subsets. The algorithm was trained on 9 subsets to learn to differentiate between honest and deceptive individuals and then validated on the remaining subset to assess its performance. This process was iterated 10 times, with each iteration featuring different training and validation subsets, guaranteeing that every data point (in this case, each of the 60 participants) is utilized for training and validation. This approach provides a comprehensive understanding of the model’s performance across subsets, minimizing the risk of overfitting and allowing for a balanced and reliable evaluation of the model’s ability to generalize.

At the end of the process, various performance metrics, such as accuracy, area under the curve (AUC, the summary of the receiver operating characteristic curve that expresses how well a model can distinguish across classes), recall, precision, and F1 score, were calculated for each algorithm to reflect the model’s overall efficacy. They are defined as follows:

1 $$Accuracy= \frac{TP+TN}{TP + FP +FN +TN}$$

2 $$Precision= \frac{TP}{TP+FP}$$

3 $$Recall= \frac{TP}{TP+FN}$$

4 $$F1 Score= \frac{2*Precision*Recall}{Precision+Recall}$$

where TP, TN FP, and FN indicate, respectively, the true positives (i.e., the number of dishonest correctly classified), true negatives (i.e., the number of honest correctly classified), false positives (i.e., the number of honest wrongly classified as dishonest), and false negatives (i.e., the number of dishonest our model did not catch) in our classification task^82^. Table 5 presents the results from the three algorithms, with logistic regression identified as the top-performing algorithm.

**Table 5:** Performance indices for the three algorithms in correctly identifying deceptive and honest participants.

Model	Performance indices				
	Accuracy	AUC	Recall	Precision	F1
Logistic regression	0.98	1.00	1.00	0.97	0.98
KNN	0.96	0.98	0.95	0.95	0.95
Random forest classifier	0.95	1.00	1.00	0.93	0.96

The table presents the values of the performance indices for the three algorithms used in the study. The indices listed across the columns are accuracy, area under the curve (AUC), recall, precision, and F1 score. The corresponding values for each algorithm are listed in the respective rows.

Further analysis revealed the frequency of false positives and false negatives across the ten folds. A false positive denotes an instance when an honest individual, per the ground truth, is erroneously identified as deceptive using the algorithm. In contrast, a false negative occurs when a deceptive individual, according to the ground truth, is incorrectly classified as honest. Across the ten folds, there was one false positive, resulting in an average rate of 0.1 for false positives and 0 for false negatives. Consequently, the logistic-regression algorithm exhibited high effectiveness with a low misclassification rate. This achievement is especially noteworthy given the relative simplicity of logistic regression as a classification method. It delivered robust results, bypassing the need for more intricate approaches, such as the use of support vector machines, Bayesian methods, and deep neural networks.

**Figure 3:**
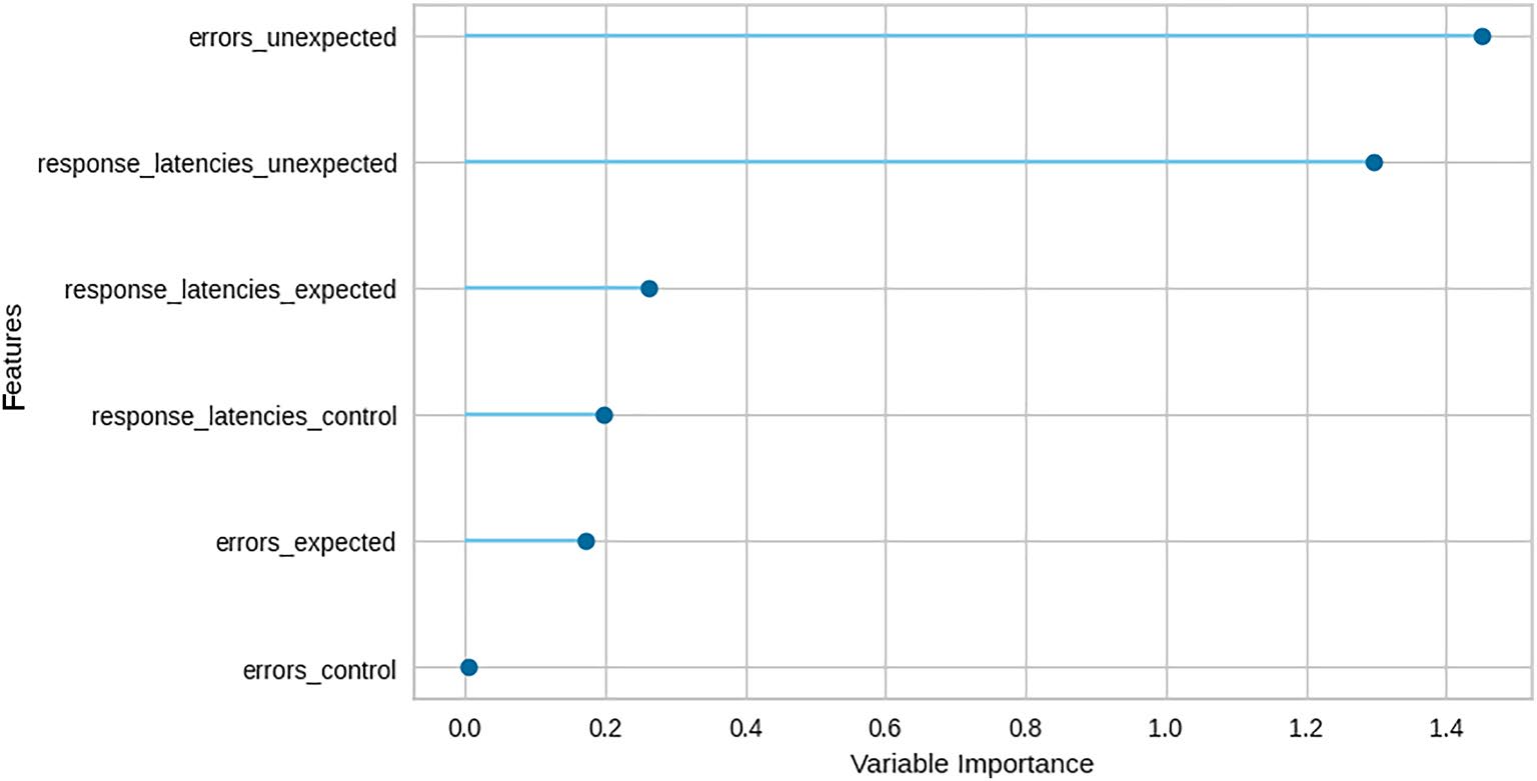
Feature importance plot. The x-axis quantifies the importance of each variable, reflecting its influence on the predictive model. The y-axis lists the six input variables in descending order of importance, from the most influential to the least.

The feature-importance plot, illustrated in Fig. 3, delineates each input variable’s impact on the logistic regression’s predictions. This visual representation helps determine which factors most significantly influence the predictions and provides insights into the interplay among variables. Notably, the errors in the answers to unexpected questions were the most indicative variable of honest and deceptive individuals, with response latencies to unexpected questions being a close second.

### Deception detection in single faked answers

Despite the notably high accuracy in distinguishing between truthful and deceptive subjects, the scenario in which a guilty suspect lies when answering all questions is exceedingly rare in investigative contexts. Typically, a guilty individual tends to lie primarily in response to directly incriminating questions, thereby complicating the detection process. Nonetheless, adopting machine-learning algorithms allows for the estimation of probabilities for accurately categorizing each answer to unexpected question as either truthful or deceptive. With the K-fold cross-validation method, response latencies and error rates of answers to unexpected questions (for the honest and deceptive groups) were used as the input variables for training and testing the three machine-learning models in categorizing each answer as honest or deceptive. Table 6 details the model’s performance metrics.

**Table 6:** Performance indices for the three algorithms in classifying single unexpected-faked answers.

Model	Performance indices				
	Accuracy	AUC	Recall	Precision	F1
Logistic regression	0.74	0.85	0.64	0.81	0.71
KNN	0.74	0.81	0.73	0.75	0.74
Random forest Classifier	0.71	0.79	0.70	0.71	0.70

The table presents the values of the performance indices for the three algorithms used in the study. The indices listed across the columns are accuracy, area under the curve (AUC), recall, precision, and F1 score. The corresponding values for each algorithm are listed in the respective rows.

This outcome demonstrates that the proposed methodology is capable not only of identifying the individual subject but also of detecting deception in each response the deceiver gives, albeit with a lower level of accuracy.

### Relative measures

The method proposed here differs from other lie detection techniques because it can be applied offline to audio recordings of investigative interviews. However, it is expected to encounter subjects from various age groups in practical applications. Considering the well-documented phenomenon that latencies and errors tend to increase with advancing age^83–85^ and the fact that our group of participants did not include all age groups, it is important to evaluate the accuracy in identifying deception by computing indices that compare the responses to unexpected questions with the responses to expected and control questions within subjects.

Such indices are particularly valuable because they diminish participant variance by comparing the subjects against themselves, using a subject-specific baseline (e.g., response latencies to control questions). This process may still accurately identify deceptive responses, albeit with a slight reduction in accuracy. For example, we incorporated additional measures, which we can designate as relative, by computing all conceivable data combinations. In this procedure, for each participant, the mean of one latency is subtracted from the mean of another, and the outcome is subsequently divided by the mean of a third latency. The same procedure was employed to compute the relative measure using errors. The list with all the tested possible combinations is reported in the Supplementary Material (S3). Subsequently, these variables were employed to classify our participants using the identical procedure detailed in the preceding paragraph. The outcomes reported in Table 7 revealed the KNN is the top-performing algorithm.

**Table 7:** Performance indices for the three algorithms using the relative measures.

Model	Performance Indices				
	Accuracy	AUC	Recall	Precision	F1
KNN	0.83	0.87	0.85	0.84	0.83
Random forest classifier	0.79	0.88	0.80	0.81	0.78
Logistic regression	0.77	0.84	0.75	0.79	0.75

The table presents the values of the performance indices for the three algorithms used in the study. The indices listed across the columns are accuracy, area under the curve (AUC), recall, precision, and F1 score. The corresponding values for each algorithm are listed in the respective rows.

Furthermore, an analysis was conducted to ascertain each feature’s impact on the classification task, which the analysis unveiled as response latencies of control-unexpected/control, denoting the result of the response latencies to control questions subtracted from that of unexpected questions and then divided by the response latencies to control questions. Employing the same machine-learning procedure, this single variable reaches very similar results to those previously obtained, yielding an accuracy rate of 81% for the top-performing algorithm (Table 8).

**Table 8:** Performance indices for the three algorithms only using the most informative relative measures.

Model	Performance indices				
	Accuracy	AUC	Recall	Precision	F1
Logistic regression	0.81	0.92	0.80	0.84	0.80
KNN	0.81	0.82	0.75	0.88	0.79
Random forest Classifier	0.70	0.79	0.70	0.68	0.67

The table presents the values of the performance indices for the three algorithms used in the study. The indices listed across the columns are accuracy, area under the curve (AUC), recall, precision, and F1 score. The corresponding values for each algorithm are listed in the respective rows.

## Discussion

### Between-subjects results discussion

This study was conducted to evaluate the credibility of responses obtained during face-to-face investigative interviews by analyzing response latencies and error rates in recorded audio. The analysis conducted by comparing how the two groups (honest and deceptive) answered unexpected questions revealed that deceptive participants exhibited prolonged response latencies and a higher frequency of errors than their honest counterparts. These findings corroborate earlier research^71,72^ and the broader literature, suggesting an augmented cognitive burden borne by individuals engaged in deceit that disrupts their ability to sustain the deceptive act^32–35^.

Additionally, we identified distinct differences between honest and deceptive responses, even when answering to expected questions, marking a departure from some of Monaro’s findings, in which no significant disparities in response times and error rates were observed^71,72^. This discrepancy could be attributed to the content of the identity information that was provided to participants (for instance, in Monaro’s expected questions, specific details, such as the initial six digits of a tax code and an email address, were omitted) and the different experimental procedure; indeed, Monaro employed mouse tracking rather than audio recording to collect response latencies. Therefore, deceptive participants demonstrated variances when deception was a factor in expected and unexpected questions.

Notably, deceptive participants did not significantly differ from honest ones in terms of error rates when they responded truthfully to control questions, corroborating the results reported in the literature^72,86^. However, we found an interesting difference between the two groups in response latencies when they answered control questions. Specifically, deceptive participants exhibited longer response latencies than honest participants, even when responding to control questions. According to the literature^87^, this effect could be explained by the task switching phenomenon. In our study, the questions were presented in random order and deceptive participants were requested to lie in response to expected and unexpected questions while being honest in response to control ones. This, in turn, introduces an additional cognitive element into the response process. The continuous cognitive and strategic switching forced upon identity deceivers influences their task performance, leading to increased response latencies, even in response to control questions.

### Within-subjects results discussion

Our results also indicated that individuals feigning their identity exhibited significant discrepancies when responding to control and expected questions versus the answers provided to unexpected questions, with the latter consistently eliciting longer response latencies and more errors. These findings are consistent with the study by Monaro et al.^53^, further corroborating our observations. Such outcomes are based on the theoretical framework of the unexpected-questions technique. This approach suggests that unexpected questions are particularly effective in eliciting deception cues from deceivers, as these questions prevent the possibility of premeditating deceptive responses^22,40,49–53,56,71,74,88,89^. Despite no difference in response latencies emerging for the deceptive group when we compared responses to control questions with expected ones, this group exhibited more errors in response to expected questions than in response to control questions. This, again, suggests an increased cognitive load for questions requiring participants to lie.

The analysis conducted on the honest group indicates that unexpected questions also impact them, resulting in prolonged response latencies and higher error rates due to the greater cognitive demand, thus supporting the literature’s findings^53^. However, the interaction effect revealed significant differences in how truth tellers and liars manage these unexpected questions. Specifically, although both groups experienced an increase in cognitive load in response to unexpected questions, resulting in longer response times and more errors compared to the control and expected questions, honest participants still exhibited shorter reaction times and fewer errors than deceptive participants. This may be attributed to the fact that they rely on actual memories rather than constructing fabricated responses.

However, the number of errors and response latencies observed when comparing answers to control and expected questions do not show any statistically significant. This lack of difference may occur because, for honest participants, responding to expected questions does not require additional effort compared to control questions, as responses must be truthful for both.

### Questionnaire results discussion

Error rates and results of response latencies analysis are corroborated by participant perceptions of the interview’s difficulty and anticipation. Participants engaging in deception reported a greater challenge in addressing expected and unexpected questions than their honest counterparts, mirroring the outcomes of Parkhouse and Ormerod^36^, in which deceptive subjects perceived the interview questions as more difficult regardless of the question type (expected or unexpected). Although the deceptive participants took significantly longer than their honest counterparts to respond to control questions, we detected no significant differences between groups in the perceived difficulty of control questions. Both groups rated answering to unexpected questions as considerably more difficult than answering to control and expected questions.

However, a notable divergence between the experimental groups emerged in their mental taxation when they responded to expected rather than control questions. Deceivers reported a greater cognitive challenge in answering to expected questions, a complexity not paralleled in the honest group, who exhibited no significant difference in perceived difficulty between these question types. The perceived increased cognitive load for deceivers responding to expected questions compared to control questions may be attributed to the necessity of reconciling known truths with the fabrication of their responses, introducing an additional layer to the response formulation process^90^.

The results from the anticipation questionnaire demonstrate that the groups do not differ in their level of anticipation for unexpected questions, thereby showing that for both groups, these were indeed unexpected. The results reveal that honest participants perceived less anticipation in answering to expected questions than the deceptive group. However, the answers to expected questions for both groups were more anticipated than the answers to unexpected questions.

### Machine-learning results discussion

In conclusion, we assessed machine-learning models’ ability to differentiate between individuals who provide deceptive responses regarding their identity and those who are truthful. We selected machine learning due to its demonstrated proficiency in classifying subjects individually, which aligns with our research objectives and the imperatives of forensic applications. Traditional statistical methods often focus on group-level variations and may lack the precision required for individual-case analysis. In contrast, machine-learning models offer a robust framework for detailed assessments of an individual’s likelihood of being truthful or deceptive—a crucial aspect in the forensic domain, in which individual accuracy at the single-subject level is required^80^.

As Table 5 shows, our models attained an accuracy rate of 98%, with a mean of only 0.1 false positives and 0 false negatives. The two most indicative features for discriminating between truth tellers and deceivers were the frequency of errors and the delays in answering unexpected questions. Furthermore, our methodology demonstrated an accuracy rate of 74% in distinguishing between deceptive and honest answers to unexpected questions. This makes it possible to determine whether the single answer the subject gave to an unexpected question is a lie or true.

### Limitations and directions for future research’

Notwithstanding the successful execution of our experiment, it is crucial to acknowledge and address its limitations. The demographic constraints of the participant pool, exclusively comprising university students aged between 21 and 29 years, limit our findings' generalizability to a broader population. Researchers should investigate these dynamics in older and younger populations. Additionally, the laboratory setting may have affected participants’ propensity for deception. Unlike real-world forensic interviews, in which the consequences of being caught are significant, the low-risk environment of a laboratory may not compel participants to employ their most convincing deceptive strategies. Researchers should consider incentivizing participants for deception to emulate more realistic high-stakes conditions. Supporting this notion, Vrij et al.^38^ found that a lie’s complexity is related to the cognitive load involved; therefore, participants might exhibit more pronounced cues of deception under higher stakes. Therefore, researchers should explore the impact of increased stakes on deceptive behavior. Another notable limitation was the exclusive use of a single experimenter for conducting and coding the audio recordings of the interviews (excluding errors coding of expected and unexpected questions). Employing a double-blind methodology would enhance the results’ credibility and mitigate potential biases. In conclusion, it is essential to recognize that posing unexpected questions is not universally applicable. This situation may arise because there may simply be no unforeseen questions that can be appropriately asked concerning the subject of the investigation. Indeed, in some instances, the nature of the topic under discussion might not accommodate the possibility of unexpected questions.

Identifying such limitations is essential for improving experimental designs and directing subsequent inquiries. Researchers should aim to replicate and extend this study’s results using a more diverse and representative sample encompassing various ages and sociocultural backgrounds. Additionally, investigating the effectiveness of the unexpected-questions technique in scenarios in which deceivers make only minor alterations to their actual biographical information, as commonly occurs^91^, would be beneficial. Moreover, the current study's results and those in the existing literature offer promising insights into the efficacy of the technique under investigation, highlighting its applicability in online and face-to-face interview settings. This is particularly relevant in an era when digital interactions are becoming increasingly prevalent, providing valuable insights to optimize interview strategies. Due to these practical implications’ importance, further exploration of this technique and the cues employed is recommended as well as further research to assess its effectiveness in a broader range of contexts.

## Conclusion

This study highlights the considerable promise of employing audio recordings and analysis of response latencies and error rates to improve deception detection in forensic interviews. This technique addresses a significant gap in the existing scientific literature by offering a method that ensures that.

1. The individual being assessed is unaware that the technique is focused on credibility evaluation.
2. The evaluation process does not disrupt the flow of investigative interviews.

Although individuals may be aware that their spoken responses are being captured, they frequently do not realize that their response latencies, subtle as they may be, can convey critical information, particularly in response to unexpected questions. This is due to the observation that response latencies and error rates when answering expected questions closely resemble those for control questions. In contrast, the latencies and error rates associated with unexpected questions provide valuable insights, proving to be an effective clue of lying because, unlike verbal content, it is harder to manipulate.

Our study shows that these measurements yield valuable insights, revealing cognitive discrepancies between honest individuals and those who are not, as the differences in response latencies and error rates show. We focused explicitly on detecting false identities, a widespread and concerning type of deception. In today’s digital era, with the ease of obtaining counterfeit documents and the increasing manipulation of personal attributes, such as images, names, and birth dates, the issue of false identity is pressing and necessitates urgent attention due to its association with various criminal activities and security breaches.

The results indicate that those impersonating others demonstrate significantly longer latencies and higher error rates than their truthful counterparts when answering unexpected questions that catch them unprepared. This difference highlights the increased cognitive burden on deceivers, who must quickly fabricate responses on the spot without premeditated replies. Their lack of preparedness for unforeseen inquiries affects their performance adversely. The study confirms that leveraging this vulnerability exposes distinct implicit cues indicative of deceit. Additionally, by randomly interspersing control, expected, and unexpected questions, we found that deceivers are forced to endure an added cognitive load as they oscillate between honesty and deception.

In forensic environments, where discerning the truth is critical, this method’s potential is evident. Although overt verbal cues can be consciously controlled, involuntary latencies serve as more stable and dependable markers of veracity. Investigators can track these time stamps via audio recordings, thus gaining a window into the individual's cognitive processes and their account’s veracity. Considering the complexities associated with deceit in legal settings, this methodology is a scientifically based tool for distinguishing between truthfulness and deception. This investigation is an initial step toward creating more sophisticated defenses against the growing menace of identity fraud.

### Supplementary Information

Supplementary Information.

## Data Availability

The datasets generated and/or analyzed during the current study are available in the “Analyzing Latencies and Errors to Unexpected Questions in Interviews for Identity Deception Detection” repository: https://osf.io/r5z67/?view_only=02f5d6a6203445f6999b74a21f8b39c1.
