# Supplementary material for: Detecting lies in investigative interviews through the analysis of response latencies and error rates to unexpected questions
Source: Sci Rep. 2024 May 28;14:12268. doi: 10.1038/s41598-024-63156-y (PMC11133341; doi:10.1038/s41598-024-63156-y)
Supplement: Supplementary file 1 — Supplementary Information. [file 41598_2024_63156_MOESM1_ESM.pdf]

Detecting Lies in Investigative Interviews Through the Analysis of Response Latencies and Error Rates to Unexpected Questions

Melis Giulia* [1,2], Ursino Martina[1], Scarpazza Cristina[1,3], Zangrossi Andrea[1,4], Sartori Giuseppe[1]

[1] *Department of General Psychology, University of Padua, Italy*

[2] *Human Inspired Technology Research Centre, University of Padua, Italy*

[3] *Translational Neuroimaging and Cognitive Lab, IRCCS San Camillo Hospital, Venice, Italy*

[4] *Padova Neuroscience Center (PNC), University of Padua, Italy*

Supplementary Materials

S1: *Instruction and False Identities Provided to the Deceptive Participants*

Instruction: *"You will now read some personal data belonging to another person, and I ask you to memorize them as best as you can. For memorization, you will have five minutes at your disposal, and I will notify you when the time is up. At the end of the five minutes, you will have to perform five arithmetic operations, and at the end of these, you will have to orally repeat the personal information. If the personal data is recalled without any errors, the next phase will begin. If you make mistakes during the recall or are unable to recall the information, you will have to repeat the previous two phases until you can recall the information without errors. Once the memorization and recall phases are concluded, you will be asked to answer some questions about the identity that was provided to you, pretending that the personal information you memorized is your own. The interview will be audio-recorded."*

Table S1. *Fictitious Identity Details*

Information	False Female Identity	False Male Identity
Name	Ginevra	Paolo
Surname	Pisani	Ricciarelli
Date of Birth	13/09/1989	28/07/1978
Place of Birth	Parma	Biella
E-mail	ginevrapisani89@hotmail.com	paoloricciarelli78@hotmail.com
Phone Number	3249168584	3313765840
First Six Letters of the Tax Code	PSNGVR	RCCPLA
Place of Residence	Salerno	La Spezia
Address	Via Porta Catena, 31	Via Fontevivo, 14
Completed Degree Course	Mathematical Sciences	Geological Sciences
Year of Degree	2011	2001

Note. The table shows the fictitious identity provided to the deceptive group to be memorized. Two identities were provided: one male (assigned to deceptive man) and one female (assigned to deceptive woman).

S2: ***Standardized Order of Questions***

At the end of the sentences, the initials "C," "E," or "U" enclosed within parentheses categorize the question as either control, expected, or unexpected, respectively.

1. What is the capital city of your region of birth? (U)
2. Where were you born? (E)
3. What is the color of the shirt you are wearing? (C)
4. What is the capital city of your region of residence? (U)
5. What is your residential address? (E)
6. In which city do you reside? (E)
7. What is your zodiac sign? (U)
8. How many odd numbers are in your date of birth? (U)
9. In which region of Italy are you currently located? (C)
10. What are the last three digits of your phone number? (E)
11. What year is it? (C)
12. What color are your eyes? (C)
13. What is your first name? (E)
14. In which month were you born? (E)
15. How old are you? (U)
16. What are your initials? (U)
17. What undergraduate degree did you complete? (E)
18. What are the first three digits of your phone number in reverse order? (U)
19. In which season are we currently? (C)
20. In which year were you born? (E)
21. What is your gender? (C)
22. What color are your shoes? (C)
23. What is your email address? (E)
24. In which year did you earn your bachelor's degree? (E)
25. How tall are you? (C)
26. What shoe size do you wear? (C)
27. In which region were you born? (U)
28. How old were you in year [add year] when you earned your bachelor's degree? (U)
29. In which month are we currently? (C)
30. Name two regions that border the region where you reside. (U)

31. What is your last name? (E)
32. What color is your hair? (C)
33. Is your birthdate closer in time to Christmas or Easter? (U)
34. How old will you be in 2025? (U)
35. In which city are you located at this moment? (C)
36. What are the first six letters of your tax identification code? (E)

S3: *Relative Measures List*

List of combinations employed in the analysis as relative measures. Each was computed by subtracting the mean of one latency from the mean of another, and the resulting value was then divided by the mean of a third latency. The same procedure was employed for errors.

1. (control-expected)/expected,
2. (control-expected)/control,
3. (control-unexpected)/control,
4. (control-unexpected)/unexpected.
5. (expected-control)/control,
6. (expected-control)/expected,
7. (expected-unexpected)/expected,
8. (expected-unexpected)/unexpected,
9. (unexpected-control)/control,
10. (unexpected-control)/unexpected,
11. (unexpected-expected)/expected,
12. (unexpected-expected)/unexpected.
